# An unexpected therapeutic overlap: long-term control of KCNK3-associated pulmonary arterial hypertension during treatment of systemic hypertension—a case report

**DOI:** 10.1093/ehjcr/ytag679

**Published:** 2026-09-15

**Authors:** Leonardo Carli, Michele di Leo, Federico Donato, Chiara Barozzi, Fabio Dardi

**Affiliations:** Department of Medical Biotechnologies, Division of Cardiology, University of Siena, Via Mario Bracci 16, Siena 53100, Italy; Department of Medical and Surgical Sciences (DIMEC), Alma Mater Studiorum, University of Bologna, Via Massarenti 9, Bologna 40138, Italy; Department of Medical and Surgical Sciences (DIMEC), Alma Mater Studiorum, University of Bologna, Via Massarenti 9, Bologna 40138, Italy; Department of Medical and Surgical Sciences (DIMEC), Alma Mater Studiorum, University of Bologna, Via Massarenti 9, Bologna 40138, Italy; Cardiology Unit, IRCCS Azienda Ospedaliero-Universitaria di Bologna, Via Massarenti 9, Bologna 40138, Italy

**Keywords:** Pulmonary arterial hypertension, KCNK3 mutation, Heritable pulmonary arterial hypertension, Vasoreactivity testing, Calcium channel blockers, Case report

## Abstract

**Background:**

Pulmonary arterial hypertension (PAH) is a progressive disorder characterized by obliterative pulmonary vascular remodelling. Pathogenic variants in KCNK3 represent a recognized cause of heritable PAH (hPAH). Loss-of-function mutations lead to membrane depolarization, increased calcium influx, and enhanced pulmonary vasoconstriction. Calcium channel blockers (CCBs) are recommended for patients demonstrating a positive acute vasoreactivity test.

**Case summary:**

A 50-year-old man carrying a pathogenic KCNK3 (E34K) mutation was referred to a tertiary PAH centre during familial screening after two of his children were diagnosed with PAH and were non-responders to acute vasoreactivity testing. He was mildly symptomatic, World Health Organization functional class (WHO-FC) II, with preserved exercise capacity, 6-min walk distance (6MWD) of 580 m, and normal NT-proBNP levels. Echocardiography showed mild right ventricular dilatation with preserved systolic function. Right heart catheterization (RHC) confirmed mild pre-capillary pulmonary hypertension with a positive acute vasoreactivity testing response. Considering the mild haemodynamic impairment, the underlying KCNK3 mutation, and prior clinical improvement during amlodipine therapy for systemic hypertension, high-dose amlodipine was initiated. At 6-month follow-up, the patient improved to WHO-FC I, with increased 6MWD and haemodynamic improvement on repeat RHC.

**Discussion:**

This case describes a favourable clinical and haemodynamic response to CCB therapy in KCNK3-associated PAH. It highlights phenotypic variability within familial KCNK3-related disease and indicates that longitudinal data may help identify patients who could benefit from targeted therapies despite borderline haemodynamic profiles.

Learning points

- Acute vasoreactivity testing should be considered in patients with heritable pulmonary arterial hypertension, regardless of the underlying genetic mutation.
- Significant phenotypic variability may occur in heritable pulmonary arterial hypertension, even among individuals carrying the same pathogenic mutation.

## Introduction

Pulmonary arterial hypertension (PAH) is a disease characterized by progressive pulmonary vascular remodelling. Several genes play a key role in heritable forms (hPAH). Among these, KCNK3 encodes a two-pore domain potassium channel regulating resting membrane potential in pulmonary arterial smooth muscle cells. Loss-of-function variants result in membrane depolarization, increased calcium influx, enhanced vasoconstriction,^1^ and vascular remodelling through proliferation of smooth muscle cells, endothelial cells, and adventitial fibroblasts.^2^

Calcium channel blockers (CCBs) are reserved for patients who demonstrate a positive response during acute vasoreactivity testing, defined as a reduction in mean pulmonary arterial pressure (mPAP) ≥ 10 mmHg to an absolute value ≤40 mmHg, with unchanged or increased cardiac output (CO).^3^ Such a response is observed in ∼12% of patients with idiopathic PAH or PAH associated with drugs and toxins, whereas it is less common (<5%) in hPAH.^4^ Although the recommendation to perform vasoreactivity testing has been extended to all patients with hPAH, the available evidence in this setting is mainly derived from patients carrying BMPR2 mutations.^5^

To the best of our knowledge, we report the first case of KCNK3-associated PAH demonstrating clinical and haemodynamic response to CCBs.

## Summary figure

**Figure:**
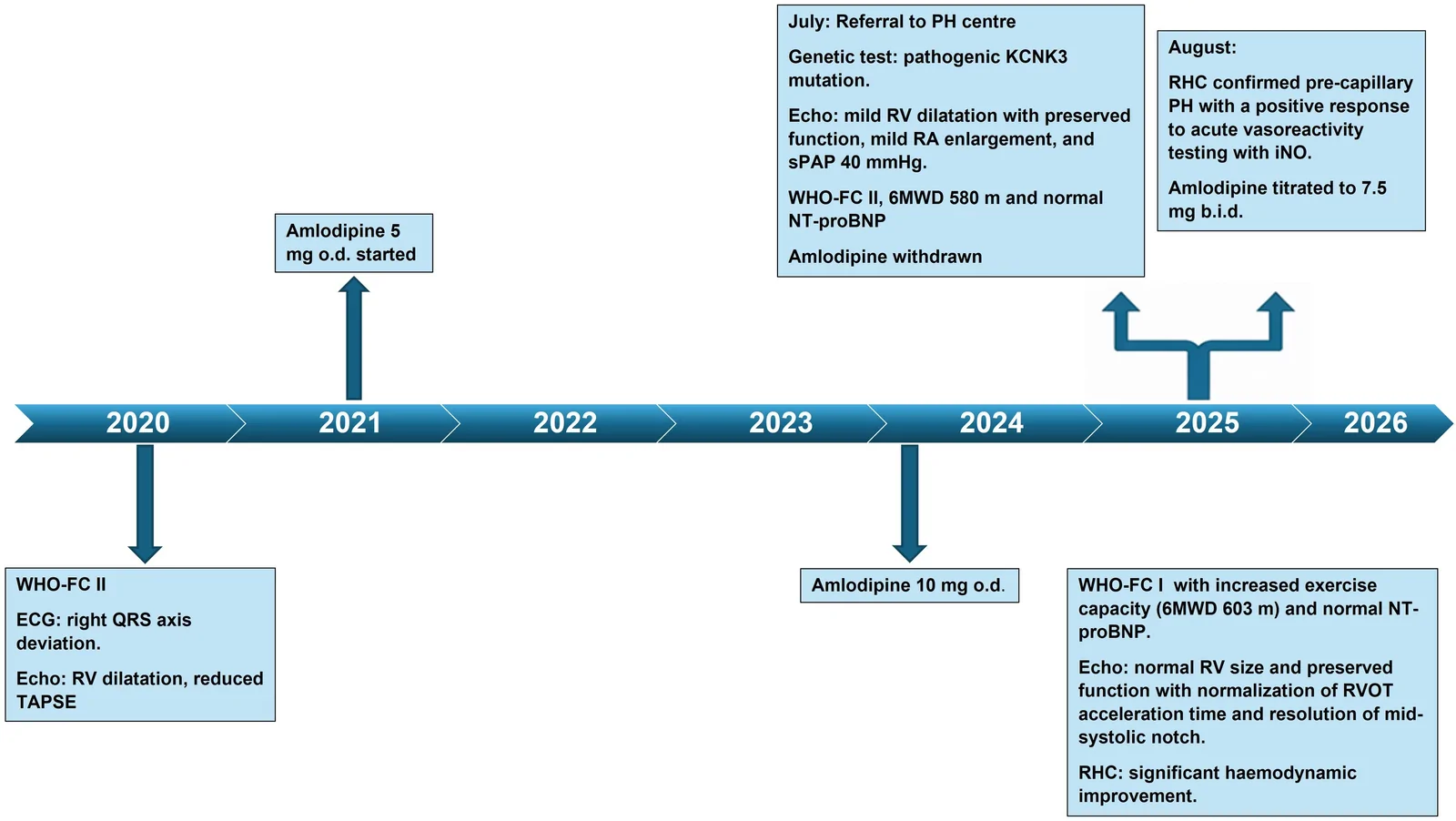


## History of presentation

In July 2025, a 50-year-old man was referred to a tertiary pulmonary hypertension (PH) centre. He is the father of two children with PAH carrying a pathogenic KCNK3 heterozygous missense (p.Glu34Lys) variant, which was also identified in him. Targeted next-generation sequencing (NGS) using a PAH-associated gene panel (82.12 kb coverage) on the Ion PGM platform (Thermo Fisher Scientific) was initially performed on his daughter (the index patient), leading to the variant’s identification. Subsequent Sanger sequencing confirmed the same KCNK3 variant in the father. The variant affects the N-terminal cytoplasmic domain of TASK-1, leading to loss of function and impaired membrane potential regulation.^1^

His medical history included systemic arterial hypertension and a 30 pack-years smoking history. Home therapy was telmisartan 80 mg o.d., hydrochlorothiazide 12.5 mg o.d., amlodipine 5 mg o.d., and carvedilol 12.5 mg b.i.d.

At referral, he was in World Health Organization (WHO) functional class (FC) II, with a 6-min walk distance (6MWD) of 580 m, normal N-terminal pro-brain natriuretic peptide (NT-proBNP) 47 pg/ml, blood pressure 115/70 mmHg, and SpO_2_ 97% on room air.

**Figure 1:**
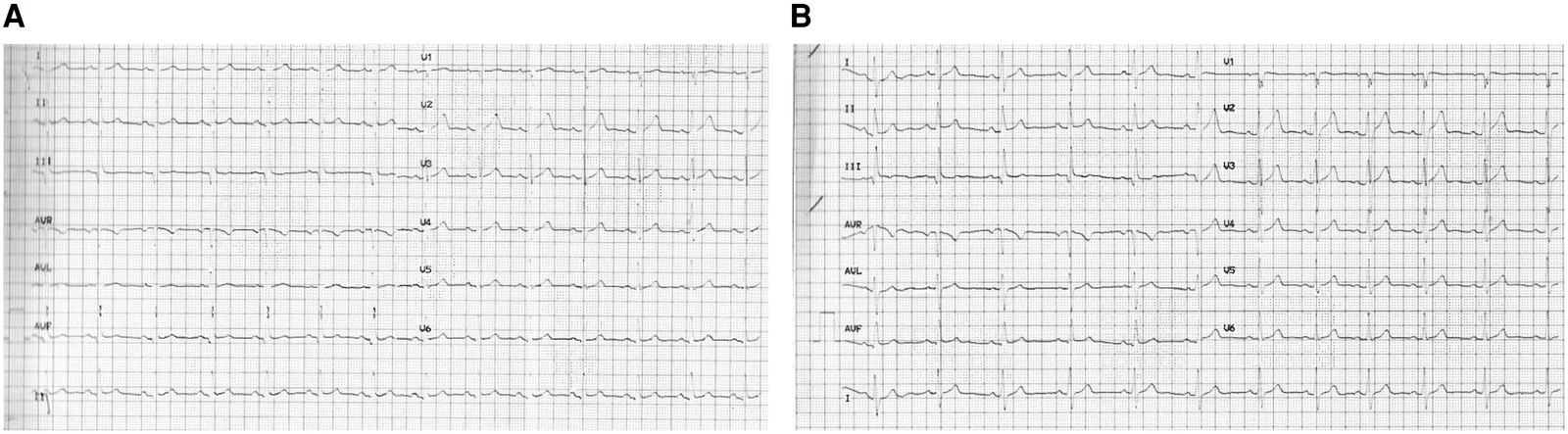
Comparison of the ECG performed in July 2024 (*A*) and at referral in July 2025 (*B*), showing normalization of QRS axis.

Electrocardiography (ECG) demonstrated a vertical QRS axis (*Figure 1*). Echocardiography revealed mild right ventricular (RV) dilatation with preserved systolic function [RV end-diastolic area (RVEDA) 25 cm^2^, RV fractional area change (RVFAC) 42%, TAPSE 2.7 cm, S’ 11.7 cm/s], reduced RV outflow tract acceleration time (RVOT-AT 95 ms) with mid-systolic notch, mild tricuspid regurgitation with estimated systolic pulmonary arterial pressure (sPAP) of 40 mmHg, and mild right atrial enlargement (19 cm^2^). Left ventricular (LV) dimensions, mass and contractility were normal (LV end-diastolic volume 40 ml/m^2^, LV mass 74 g/m^2^, LV ejection fraction 65%), as was left atrial volume (32 ml/m^2^).

Prior records showed longstanding abnormalities. In 2020, the patient presented with atypical chest pain; coronary angiography excluded coronary artery disease. ECG showed right QRS axis deviation, and transthoracic echocardiography demonstrated RV dilation with mildly reduced longitudinal function (TAPSE 15 mm). The patient declined further investigations. No clinical events occurred thereafter. A follow-up echocardiogram in January 2021 confirmed RV dilatation (RV–to–LV basal ratio 1.2), right atrial enlargement (21 cm^2^), and mild tricuspid regurgitation with estimates of sPAP of 35 + 5 mmHg.

Amlodipine 5 mg o.d. had been taken irregularly since January 2021. In July 2024, the dose was increased to 10 mg o.d. for suboptimal blood pressure control; ECG at that time showed right QRS axis deviation (*Figure 1*). The patient reported subjective improvement in dyspnoea after dose escalation. In June 2025, the dose was reduced again to 5 mg o.d. due to seasonal blood pressure control.

## Investigations

A comprehensive PH work-up was performed.

**Figure 2:**
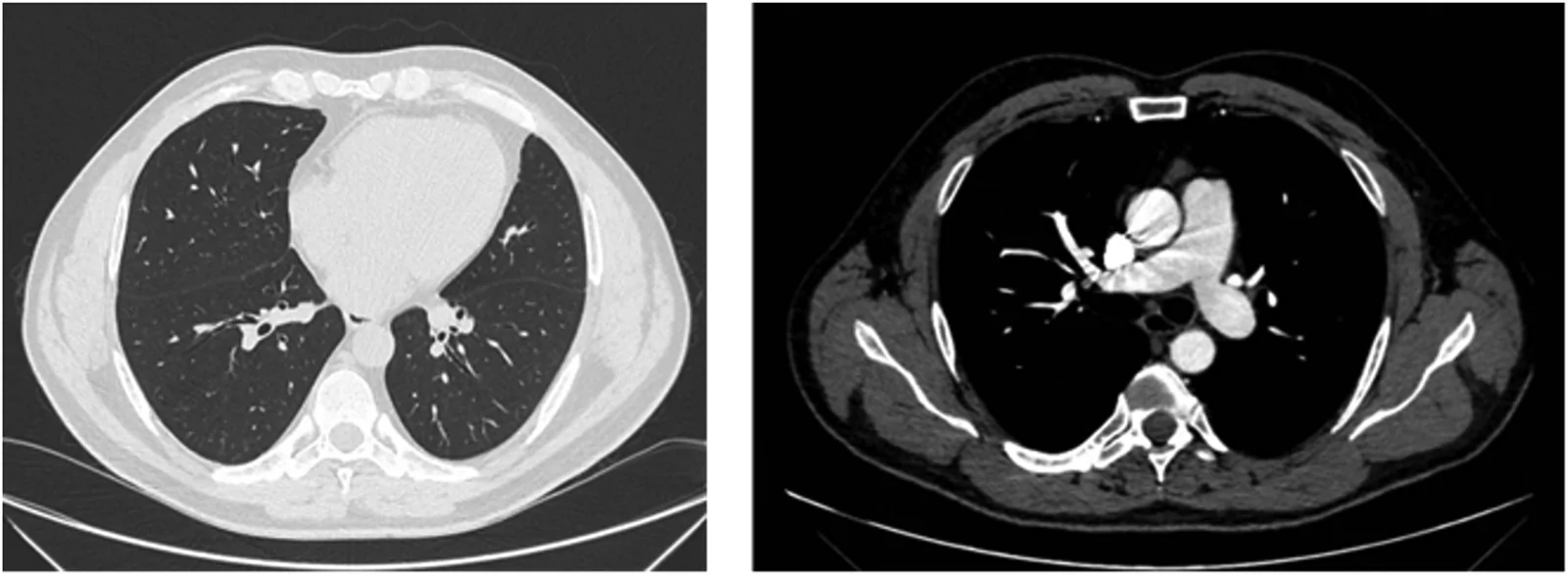
Chest computed tomography documenting normal lung parenchyma and absence of thromboembolic disease of the pulmonary vasculature.

High-resolution computed tomography (CT) and CT pulmonary angiography showed no evidence of parenchymal abnormalities. The pulmonary arteries were not dilated, and no thromboembolic defects were identified (*Figure 2*).

Lung function tests, including diffusion capacity for carbon monoxide (DLCO), were within normal limits [TLC 6.3 L (91%), RV 1.6 L (73%), FVC 4.7 L (106%), FEV1 4 L (111%), FEV1/FVC 86%, DLCO 10.2 mmol/(min·kPa) (101%)]. Arterial blood gas analysis was normal (pH 7.39, PaO_2_ 82 mmHg, PaCO_2_ 43 mmHg, SaO_2_ 97%, HCO_3_^−^ 26 mmol/L).

In the absence of significant parenchymal lung and/or left heart diseases, and given the pathogenic KCNK3 variant, right heart catheterization (RHC) with acute vasoreactivity testing was performed. Amlodipine was discontinued one month prior to RHC to avoid interference.

**Figure 3:**
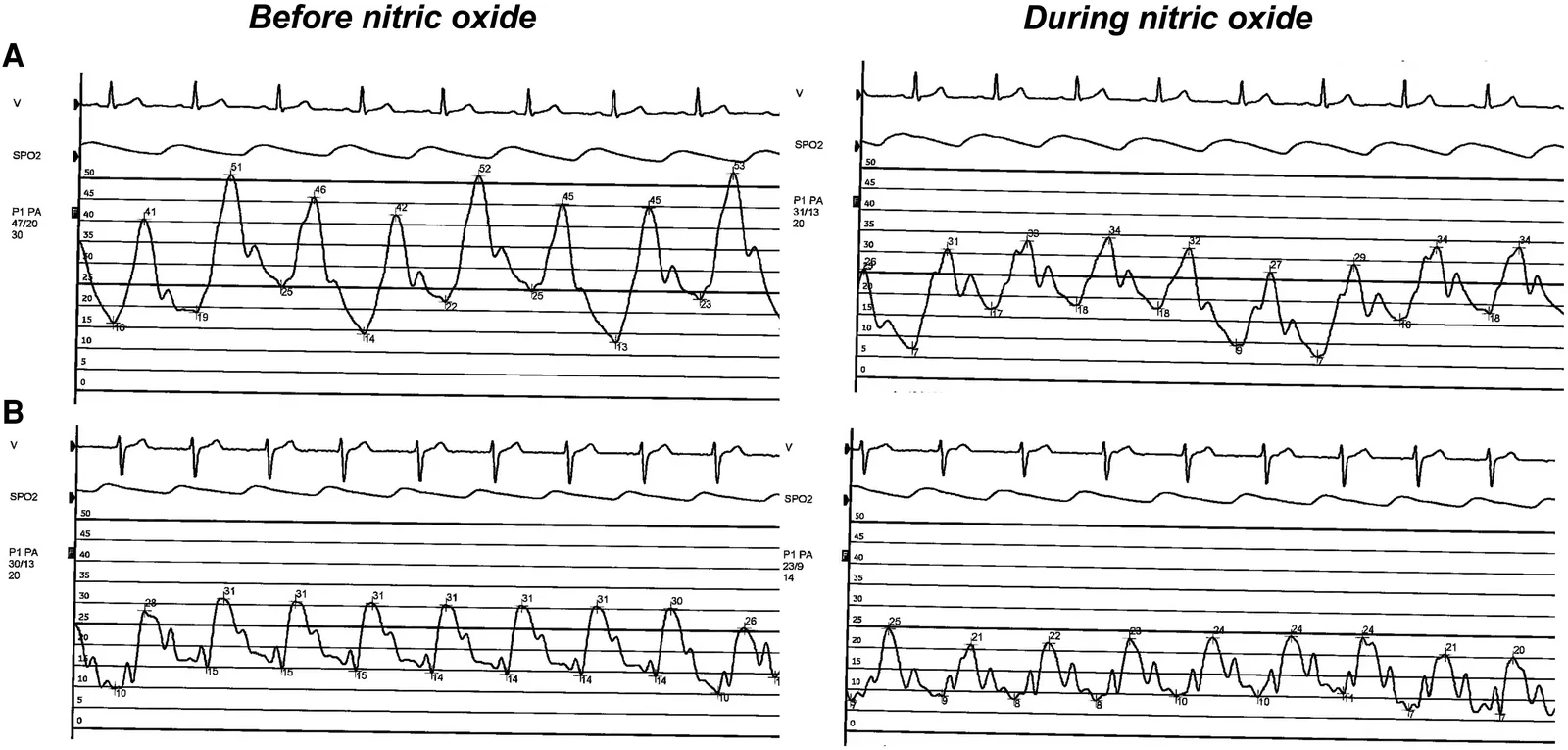
Pulmonary arterial pressure tracing during right heart catheterization at the time of referral (*A*) and after 6 months of high dose amlodipine (*B*). The left column shows the PA pressure curves before nitric oxide inhalation, the right column shows the PA pressure curves during acute vasodilator test with nitric oxide.

RHC confirmed pre-capillary PH with borderline pulmonary vascular resistance (PVR) between 2–3 WU. Acute vasoreactivity testing with inhaled nitric oxide (NO) fulfilled criteria for a positive response (*Table 1*, left panels; *Figure 3A*), with complete normalization of the pulmonary haemodynamics during NO inhalation.

**Table 1:** Haemodynamic profile at diagnosis and 6 months after initiation of high-dose amlodipine

	Diagnostic RHC		6 months follow-up	
	Before NO	During NO	Before NO	During NO
BMI (kg/m^2^)	29		27	
HR (bpm)	62	68	75	76
RAP (mmHg)	7	5	6	6
s/d/m PAP (mmHg)	47/20/30	31/13/20	30/13/20	23/9/14
s/d/m BP (mmHg)	120/72/89	142/92/113	108/63/80	117/71/88
PAWP (mmHg)	10	12	8	9
CO (l/min)	7.0	7.1	6.9	6.4
CI (l/min/m^2^)	3.4	3.4	3.4	3.2
PVR (WU)	2.9	1.1	1.7	0.8
SvO_2_ (%)	72	76	76	78
Art O_2_ (%)	99	99	99	99
Therapy at the time of RHC	Telmisartan/hydrochlorothiazide 80/12.5 mg o.d., carvedilol 12.5 mg b.i.d. ^a^		Amlodipine 7.5 mg b.i.d., telmisartan/hydrochlorothiazide 80/12.5 mg o.d., atorvastatin 20 mg o.d.	

BMI, body mass index; HR, heart rate; RAP, right atrial pressure; s/d/m PAP, systolic/diastolic/mean pulmonary arterial pressure; s/d/m BP, systolic/diastolic/mean systemic arterial blood pressure; PAWP, pulmonary artery wedge pressure; CO, cardiac output; CI, cardiac index; NO, nitric oxide inhalation; PVR, pulmonary vascular resistance; SvO_2_, mixed venous oxygen saturation; Art O_2_, arterial oxygen saturation; RHC, right heart catheterization.

^a^Amlodipine 5 mg OD had been temporarily withheld for a month before RHC.

## Management

Considering the mild haemodynamic impairment with a slowly progressive phenotype, normalization of haemodynamics during NO inhalation with formal responder criteria, pathophysiological plausibility related to KCNK3 dysfunction, prior clinical benefit with CCBs with subjective improvement in dyspnoea and serial ECGs showing progressive normalization of the QRS axis, and recent evidence suggesting treatment benefits in patients with borderline PVR without cardiac or pulmonary comorbidities explaining the haemodynamic abnormalities,^6^ high-dose amlodipine was reintroduced and gradually up-titrated to 7.5 mg b.i.d. Beta-blocker was withdrawn due to contraindication in PAH.

## Outcome and follow-up

At 6 months, the patient improved to WHO-FC I, with an increased 6MWD (603 m). NT-proBNP remained normal (47 pg/ml).

Transthoracic echocardiography showed stable findings (RVEDA 21 cm^2^, RVFAC 45%, TAPSE 25 mm, S’ 11.3 cm/s). RVOT-AT increased to 110 ms, with resolution of the mid-systolic notch.

Repeat RHC confirmed haemodynamic improvement, with normalization of mPAP and PVR (*Table 1*, right panels; *Figure 3B*) yielding a profile comparable to that observed during NO inhalation at baseline. Acute vasoreactivity testing showed a further reduction in mPAP but with decreased CO; therefore, amlodipine was not further up-titrated.

## Discussion

The relationship between vasoreactivity and KCNK3 mutations is complex. Loss-of-function variants drive hPAH, while reduced KCNK3 expression is also found in idiopathic or BMPR2-associated PAH.^7^

In rat models, genetic knockout or inhibition induces PH through enhanced vasoconstriction and distal neomuscularization—driven by proliferation of smooth muscle cells, endothelial cells, and adventitial fibroblasts.^7,8^

Consistently, histopathology in KCNK3-associated PAH reveals medial hypertrophy, fibro-intimal proliferation, and plexiform lesions.^1^ Although current guidelines recommend acute vasoreactivity testing in patients with hPAH, responsiveness has not previously been reported in KCNK3-associated forms.

A direct genotype–phenotype relationship remains hypothesis-generating. Notably, neither of the patient’s affected children, carrying the same mutation, exhibited vasoreactivity; both presented with markedly earlier disease onset (2 and 8 years), and one died at the age of 16.

This case represents, to our knowledge, the first report of discordant vasodilator responses within a familial cluster of hPAH, while also highlighting intrafamilial phenotypic variability in terms of age at onset and disease severity, and supporting guideline-recommended use of CCBs in vasoreactive hPAH patients.

## Conclusion

In this familial case of KCNK3-associated heritable PAH, CCB therapy was associated with clinical and haemodynamic improvement, reinforcing the importance of not omitting vasoreactivity testing in hPAH despite its lower expected yield. The discordant vasoreactivity observed within the same family highlights the phenotypic variability that might be associated with KCNK3-related disease. Additional cases are needed before any genotype-specific therapeutic implications can be reliably established.

## Lead author biography


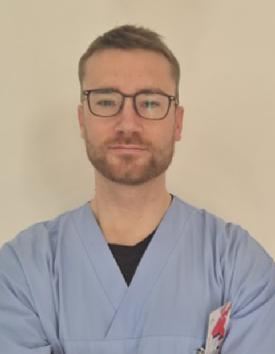


Resident in Cardiology with a special interest in heart failure, acute cardiovascular care and pulmonary hypertension. During my training, I had the opportunity to undertake a clinical training period at the Pulmonary Hypertension Center of Sant’Orsola Hospital in Bologna, where the clinical case presented in this report was identified during my work with the team

## Data Availability

The data underlying this article will be shared on reasonable request to the corresponding author
